# Association of cytokine levels with treatment duration and patient family history in Egyptian multiple sclerosis patients

**DOI:** 10.1038/s41598-026-38500-z

**Published:** 2026-03-02

**Authors:** Esraa Mohsen, Hesham Haffez, Sandra Ahmed, Taghrid S. El-Mahdy, Selwan Hamed

**Affiliations:** 1 Department of Microbiology and Immunology, Faculty of Pharmacy, Capital university, formerly Helwan University, Ain Helwan, Helwan, 11795 Egypt; 2 Department of Biochemistry and Molecular Biology, Faculty of Pharmacy, Capital university, formerly Helwan University, PO Box 11795, Cairo, Egypt; 3 Center of Scientific Excellence “Helwan Structural Biology Research, (HSBR)”, Capital university, formerly Helwan University, Cairo, 11795 Egypt; 4 Department of Neurology, Faculty of Medicine, Cairo University, Cairo, Egypt; 5 Department of Microbiology and Immunology, Faculty of Pharmacy, Modern University for Technology and Information (MTI), Cairo, Egypt

**Keywords:** Multiple sclerosis, Proinflammatory cytokines, DMTs, EDSS, Egypt, Diseases, Immunology, Neurology, Neuroscience, Rheumatology

## Abstract

Multiple sclerosis (MS) is one of the diseases that is widely spreading all over the world, with no clear etiology or definite pathological mechanism. Although there is no cure for MS, multiple therapeutic agents called disease-modifying therapies (DMTs) have been developed to relieve worsening symptoms and counteract its progression. The aim of this study is to investigate the effect of DMTs on some proinflammatory cytokine levels in the serum of Egyptian MS patients over different treatment durations. Additionally, link the levels of serum cytokines with patients’ clinical parameters. A total of 192 MS Egyptian patients were recruited and classified based on treatment duration and DMT types. The levels of IL-6, IL-17A, TNF-α, and IFN-γ were detected using the ELISA technique. Results showed that MS patients treated for a period longer than 24 months were associated with a significant decrease in IL-6, TNF-α, and IFN-γ levels compared to untreated patients or patients treated for less than 12 months. IL-6 correlated directly with the Expanded Disability Status Scale score, whereas IL-17A and IFN-γ were inversely correlated in treatment-naïve MS patients. Additionally, MS patients with a family history of autoimmunity have a lower age at onset of the disease with a higher TNF-α level. In conclusion, proinflammatory cytokine levels were correlated with MS patients on long-term treatment with DMTs. IL-6 was linked with worsening disability in MS patients, while TNF-α was linked to a family history of autoimmunity.

**Supplementary Information:**

The online version contains supplementary material available at 10.1038/s41598-026-38500-z.

## Introduction

Multiple sclerosis (MS) is a chronic inflammatory demyelinating disease involving an immune response in the pathogenesis affecting the central nervous system (CNS). This results in the slowing or blocking of nerve impulses due to partial damage or complete loss of the protective myelin sheath around nerve cells^1^. The most common symptoms of MS are limb weakness, sensory abnormalities, ocular symptoms, ataxia, and psychiatric symptoms, along with other neuronal complications that may progress to paralysis^2^.

The proposed mechanism of MS is related to the inclusion of different immune cells that are activated in the periphery and then migrate and infiltrate into the CNS, releasing proinflammatory cytokines leading to activation of CNS resident immune cells, which in turn secrete additional proinflammatory cytokines and attract further peripheral immune cells^3^. These events can induce inflammation within the CNS and promote loss of oligodendrocytes, reactive gliosis, neuro-axonal destruction, demyelination and degeneration in MS^4^. In later stages of MS, the activated microglia secreting proinflammatory cytokines are considered the main pathologic hallmark of disease progression^5^.

The diagnosis of MS is based on a combination of medical history, physical exam, magnetic resonance imaging (MRI), and spinal tap results; however, there are no specific biochemical tests for MS diagnosis. Physical tools, such as MRI, determine the presence of the active inflammatory activity (T1 lesions) or demyelinating lesion load and their distribution in the CNS (T2 lesions) along with basic cerebrospinal fluid (CSF) biochemistry to detect immunoglobulin G (IgG) intrathecal synthesis, both quantitatively (IgG index) and qualitatively (oligoclonal band [OCB] analysis)^6^. The medical history and physical exam determine the number of relapses/attacks^6^ and the Expanded Disability Status Scale (EDSS) to measure disability and progression in MS patients^7^. Moreover, the latest McDonald criteria (2024 revised criteria)^8^ for MS diagnosis recommends additional approaches aimed at speeding up the diagnosis of MS, while maintaining specificity and reducing time to treatment initiation. One of the advanced approaches is the inclusion of the Central vein sign (CVS), lesions that have a central vein^18^. Demonstration of CVS by MRI can increase diagnostic specificity by differentiating MS from other vascular or CNS inflammatory conditions^19^, and discriminate 87% of people incorrectly diagnosed with MS^9^. The other is the paramagnetic rim lesions (PRLs), chronic active MS lesions characterized by an inactive core surrounded by activated iron-laden microglia. PRLs are rarely found in other radiological mimics of MS^20^. According to a recent systematic review and meta-analysis, PRLs’ composite specificity for the MS diagnosis was 98%, while their sensitivity varied from 10% to 92.3%^21^.

In meta-analysis, various studies have demonstrated an increase of some traditional pleiotropic proinflammatory cytokines, such as IL-6, IL-17, TNF-α, and IFN-γ, in the CSF or serum of MS patients^10^. Elevated levels of IL-6 were detectable in the CSF of MS patients, which were correlated with disease activity^11^. CD4^+^ T cells secreting IL-17 were observed to be increased and correlated with active brain lesions in MS patients^12^. In experimental autoimmune encephalomyelitis (EAE), astrocytes express IL-17 receptor A, and this expression is upregulated^13^. Similarly, elevated levels of TNF-α have been observed in the central and peripheral demyelinating lesions, serum, and CSF of MS patients, confirming its role in MS pathogenesis^14^. IFN-γ is a major cytokine found in MS lesions, and it has been considered the hallmark of Th1 cells driving inflammation and autoimmunity in MS^15^. Conversely, there was evidence about the protective role of IFN-γ in reducing the severity, morbidity, and mortality in EAE^16^.

Based on the immunological etiology of MS, several therapeutic agents have been suggested to ameliorate inflammation and delay the progression of the disease, rather than being complete curative treatments for MS^17^. The FDA has authorized many types of disease-modifying treatments (DMTs) with varying efficacies for the management of MS, such as interferon beta (IFN-β), glatiramer acetate, natalizumab, fingolimod, alemtuzumab, teriflunomide, and dimethyl fumarate (DMF)^18^, and rituximab is used off-label^17^. DMTs work through different mechanisms, including reduction in the proinflammatory mediators’ secretion and immune cell hyperreactivity. Some of DMTs act as immunomodulators (e.g., fumarates), immune cell migration inhibitors, such as sphingosine-1-phosphate (S1P) receptor modulators (e.g., fingolimod), and cell-depleting monoclonal antibodies (e.g., ocrelizumab)^18^. In this study, we aimed to correlate the serum cytokine levels with the treatment durations and some demographic and diagnostic parameters of MS patients.

## Materials and methods

### Study subjects

In this study, total 192 MS patients were recruited during the past 12 months from the MS research unit, EL-Kasr Al-Ainy, Cairo University. MS patients were clinically diagnosed according to the 2017 McDonald criteria, which remain compatible with the updated McDonald 2024 criteria (a substantial revision of the 2017 McDonald)^8^. The inclusion criteria of the MS patients are (a) Egyptian patients above 18 years of age, (b) clinically definite MS (CDMS) with a clear clinical course (relapsing–remitting (RRMS), secondary progressive (SPMS)), and (c) availability of a detailed clinical and treatment history. Patients in treatment with DMTs such as IFN-β, DMF, rituximab, fingolimod, or therapies for MS symptom management were enrolled in the present study and classified into 4 different groups (gp) according to treatment duration: **treatment gp 1** includes recently diagnosed treatment naïve (RDTN) patients (without treatment until sampling), who are diagnosed within 3–6 months, **treatment gp 2** includes short-term treated patients who received treatment for less than 12 months, **treatment gp 3** includes untreated old, diagnosed patients (not receiving treatment for a period longer than the washout period [a period after stopping receiving treatment to ensure that there was no residual treatment effect]). Treatment gp 3 consists of 27 RRMS and 21 SPMS patients, who are classified according to prior DMT exposure as follows: 18 patients who had not received any DMT since their MS diagnosis for more than 4 years. Additionally, 6 patients had previously been treated with rituximab, with the last dose administered 1.5 to 3 years before study enrollment (rituximab washout period: 6–9 months^19^). Twelve patients had discontinued fingolimod between 5 months and 4 years before inclusion (washout period: 4–8 weeks^20,21^), and 12 patients stopped IFN-β between 10 months and 5 years prior to inclusion (washout period: 2–3 months^22^). Treatment gp 4 includes long-term treated patients who received treatment for more than 24 months. Patients who received corticosteroids for less than one month prior to enrollment were excluded. Ethical approval for this study was obtained according to the regulations and recommendations of the Declaration of Helsinki by the research ethics committee of the Faculty of Pharmacy, Helwan University (approval No. 08H 2018).

### Demographic and physical data collection

The detailed patient history and demographic data were retrieved during sample collection, including sex, age, medical and family history, treatments received, EDSS^7^, and clinical tests such as IgG index and CSF-restricted OCBs.

### Biochemical analysis

Blood samples were collected into serum-separating tubes (anticoagulant-free sterile tubes) and left for 15–30 min at room temperature to ensure blood clotting. Then the serum was separated by centrifugation at 1000–2000 ×*g* for 10 min and stored until analysis at − 80 °C immediately after centrifugation and aliquoting, typically within 30–45 min of collection. The serum samples were visually inspected for hemolysis, and any samples exhibiting pinkish/reddish discoloration were excluded from cytokine analysis. The serum levels of IL-6, IL-17A, TNF-α, and IFN-γ (pg/mL) were measured using enzyme-linked immunosorbent assay (ELISA) by commercial kits (Innova Biotech, China) according to the manufacturer’s procedure and previously used protocol^23^. The serum cytokine levels in healthy Egyptian controls reported to be lower than patients with different immunoinflammatory diseases, measured using the ELISA technique, as follow: for IL-6: 12.25 ± 7.90 pg/ml^24^, 21.15 ± 10.99 Pg/ml^25^, and for IL-17A: 16.63 ± 13.14^26^, 25.36 ± 5.39^27^, 55 (3.0–180.0)^28^. For TNF-α: 40.1 ± 38.2 pg/ml^29^, 76.6 (43–141)^30^, 172.7 ± 39.19Pg/ml^25^, and IFN-γ: 8.17 ± 1.96^31^, 50.91 ± 6.20^32^.

### Statistical analysis

The statistical analysis of data was performed using the GraphPad Prism 8.0 statistical software package (for Windows or Mac, Version 8.0.2, USA). The normality of continuous variable distribution was evaluated by the Kolmogorov–Smirnov test. The statistical difference between two independent groups was done using the Mann–Whitney U test or student’s t-test, while, for more than two groups, the one-way ANOVA test or Kruskal–Wallis tests were used, depending on the distribution of the data. For categorical variables, the chi-square test was used to compare the significance between groups; *P* < *0.05* was considered statistically significant.

Univariate analyses were performed using a two-way ANCOVA test in SPSS in order to examine each cytokine separately. The dependent variables were the cytokine levels (IL-6, IL-17A, TNF-α, and IFN-γ), while the independent factors were DMT class and gender. The interaction between DMT class and gender was assessed after adjusting for potential confounders, including age, disease duration, and MS phenotype.

## Results

### Demographic data

All data from the 192 patients used in the study were summarized in Table 1. Treatment classification for the patients includes treatment gp 2, who received only one DMT class from diagnosis till time of sampling, and they are classified as IFN-β (56.25%), fingolimod (18.75%), DMF (12.5%), and rituximab (12.5%). Treatment gp 4 patients received multiple classes of DMTs during their treatment period, while the current DMTs are fingolimod (50%), rituximab (18.75%), DMF (12.5%), ocrelizumab (6.25%), methotrexate (6.25%), and IFN-β (6.25%).

**Table 1:** Demographics characters of MS patients.

Demographic characteristics	MS patients (%)
Total number	192 (100%)
Age (years) (Mean ± SEM)	32.73 ± 0.7284
Females	144 (75%)
Males	48 (25%)
RRMS	153 (79.68%)
SPMS	39 (20.31%)
Treatment gp 1	48 (25%)
Treatment gp 2	48 (25%)
Treatment gp 3	48 (25%)
Treatment gp 4	48 (25%)
EDSS at diagnosis (Mean ± SEM)	3.011 ± 0.1582
EDSS at sampling (Mean ± SEM)	3.5 ± 0.2012
Number of relapses (Mean ± SEM)	2.61 ± 0.1919
Age at onset (Mean ± SEM)	26.29 ± 0.7096
Diseases duration (years) (Mean ± SEM)	6.085 ± 0.4378
IgG index (Mean ± SEM)	0.92 ± 0.04249
CSF-OCBs	Positive 72 (37.5%) Negative 12 (6.25%) ND 108 (56.25%)
Family history of MS	6 (3.125%)
Family history of autoimmune diseases	21 (10.9%)

RRMS; Relapsing remitting multiple sclerosis, SPMS; Secondary progressive multiple sclerosis, EDSS; Expanded disability status scale. CSF-OCB; CSF-Oligoclonal bands, ND: not determined

### Biochemical analysis

#### Effect of different treatment durations on serum cytokine levels

Considering the effect of treatment duration on cytokine levels, IL-17A and TNF-α levels were significantly higher in short-term treated patients (treatment group 2) than in untreated groups (treatment gp1 and treatment gp3) (*P* = *0.0239*, *0.0497*, respectively). Moreover, IL-6, TNF-α, and IFN-γ levels were significantly lower in long-term treated patients (treatment gp 4) than in untreated (treatment gp 1, 3) or short-term treated patient groups, as shown in Table 2. Notably, IL-17A levels did not show a statistically significant reduction in the long-term treatment group compared with the short-term treatment group. The levels of serum cytokines across different treatment durations in RRMS and SPMS patients are provided in Tables S1 and S2.

**Table 2:** Serum cytokine levels within different treatment durations (Groups 1 to 4; n = 48 each).

Serum cytokines (pg/ml)	Non-treated (Treatment gp 1, 3) (n = 96)	Short treated (Treatment gp 2) (n = 48)	Long treated (Treatment gp 4) (n = 48)	*P* value
	Median (IQR)			
IL-6	25.90 (21.76–32.97)	25.90 (21.84–33.48)	**#16.41 (13.22–21.5)	0.0001*
IL-17A	43.21 (26.25–58.21)	^**@**^56.07 (33.75–66.43)	47.86 (35.0–61.61)	0.0275*
TNF-α	160.8 (123.3–179.6)	^**@**^176.7 (128.7–198.8)	**#106.7 (68.3–172.1)	< 0.0001*
IFN-γ	33.50 (29.75–46.62)	34.75 (30.2–47.43)	**#29.04 (21.27–42.4)	0.0021*

^**@**^ indicates significance between Treatment gp 1,3 Vs. Treatment gp 2 (Dunn’s test).

# indicates significance between Treatment gp 1,3 Vs. Treatment gp 4 (Dunn’s test).

** indicate significance between Treatment gp 2 Vs. Treatment gp 4 (Dunn’s test).

* indicates statistical significance (*P* < 0.05) among groups.

IL; Interleukin, TNF- α; Tumor necrosis factor alpha, IFN-γ; Interferon gamma.

[kruskal–wallis test with approximate *P* value/Dunn’s multiple comparisons test using adjusted *P* value].

*P* values represent overall differences among the three groups assessed by the Kruskal–Wallis test; post-hoc pairwise comparisons (Dunn’s test) are not shown.

#### Effect of different treatment classes on serum cytokine levels

As described in Table 3, using different DMTs for a short term (less than 12 months) fails to reduce serum cytokine levels significantly; additionally, short-term rituximab treatment (4–5 months) was associated with a significant increase in all cytokine levels.

**Table 3:** Serum cytokine levels between treatment naïve patients (treatment gp 1) and short treated patients with different DMTs (treatment gp 2).

Serum cytokines (pg/ml)	Treatment gp 1	Treatment gp 2			
		IFN-β (n = 27)	Fingolimod (n = 9)	DMF (n = 6)	Rituximab (n = 6)
Median (IQR)					
IL-6	24.86 (21.5–30.55)	24.86 (22.1–29.0)	34.52 (25.21–34.86)	21.59 (21.41–21.76)	57.79 (26.59–89.0)
*P* value (against gp1)	> 0.9999	0.0352*	0.2342	0.0382*	
IL-17A	38.93 (28.21–55.89)	52.50 (31.79–58.21)	39.64 (24.64–58.21)	61.79 (54.64–68.93)	115.4 (71.79–158.9)
*P* value (against gp1)	0.9192	> 0.9999	0.1273	0.0004*	
TNF-α	152.5 (102.9–190.4)	177.5 (137.5–199.2)	125.8 (107.5–152.5)	178.3 (174.2–182.5)	298.3 (185.8–410.8)
*P* value (against gp1)	0.1033	0.8074	> 0.9999	0.0073*	
IFN-γ	33.14 (24.93–47.79)	31.71 (26.36–43.14)	31.00 (29.93–33.86)	44.04 (35.64–52.43)	66.71 (43.14–90.29)
*P* value (against gp1)	> 0.9999	> 0.9999	0.2186	0.0209*	

IL; Interleukin, TNF-α; Tumor necrosis factor alpha, IFN-γ; Interferon gamma, DMF; Dimethyl fumarate.

* indicates statistical significance (*P* < 0.05) among groups.

[Mann–whitney test using exact *P* value].

#### Univariate analysis for each cytokine

**Fig. 1:**
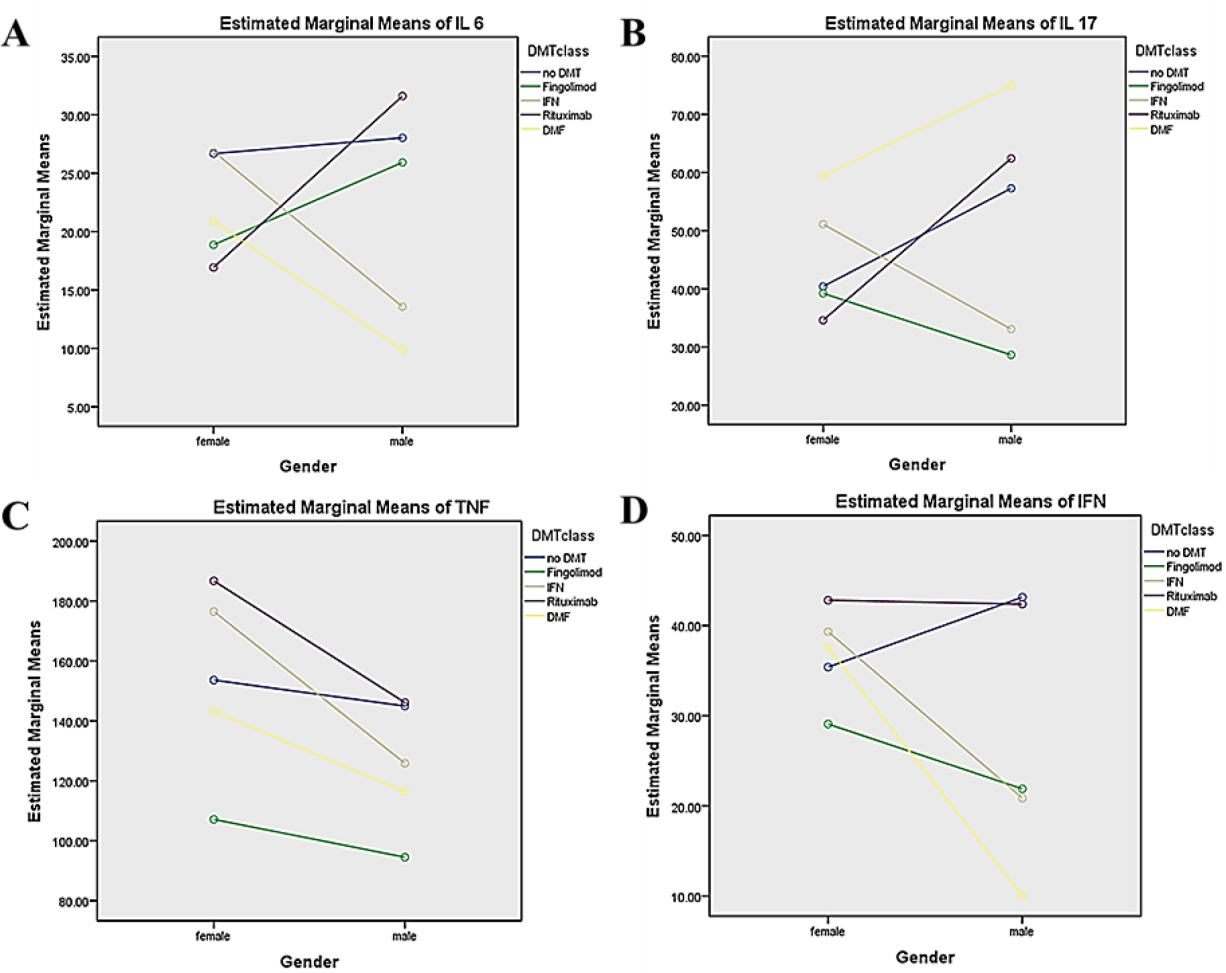
Estimated marginal means of cytokine levels by DMT class and gender (Profile Plots).The figure displays the adjusted marginal means for (**A**) IL-6, (**B**) IL-17A, (**C**) TNF-α, and (**D**) IFN-γ across different disease-modifying therapy (DMT) classes—no DMT (no treatment), fingolimod, IFN-β,Rituximab (includes patients receiving rituximab or ocrelizumab), and dimethyl fumarate (DMF)—stratified by gender. Lines demonstrate the interaction patterns between treatment class and gender, showing treatment-specific and gender-specific variations for each cytokine. Values are adjusted for age, disease duration, and MS phenotype.

The Univariate analysis revealed a significant interaction between DMT classes and gender for IL-6, IL-17A, and IFN-γ levels, but not for TNF-α. Among the significant interactions, the strongest effect was observed for IFN-γ (η2p = 0.233) and the weakest for IL-17A (η2p = 0.136) (Table 4). These findings suggest a complex interaction between DMT classes and gender in modulating cytokine levels (Fig. 1**).**

**Table 4:** The Univariate analysis for each cytokine.

Independent variables	IL-6		IL-17A		TNF-α		IFN-γ	
	*P* value	(η^2^_p_)	*P* value	(η^2^_p_)	*P* value	(η^2^_p_)	*P* value	(η^2^_p_)
DMT classes	0.00*	0.154	0.00*	0.124	0.001*	0.111	0.00*	0.266
Gender	0.898	0.000	0.111	0.016	0.015*	0.037	0.00*	0.096
DMTs * gender	0.00*	0.161	0.00*	0.136	0.512	0.021	0.00*	0.233

DMT classes groups: no DMT (without treatment), fingolimod, IFN-β, Rituximab (patients receiving rituximab or ocrelizumab), and dimethyl fumarate (DMF).

The adjusted confounders, including age, disease duration, and MS phenotype.

IL; Interleukin, TNF-α; Tumor necrosis factor alpha, IFN-γ; Interferon gamma, (η^2^_p_); Partial Eta Squared.* indicates statistical significance (*P* < 0.05).

#### Correlation of patients’ disability status with serum cytokine levels

**Fig. 2:**
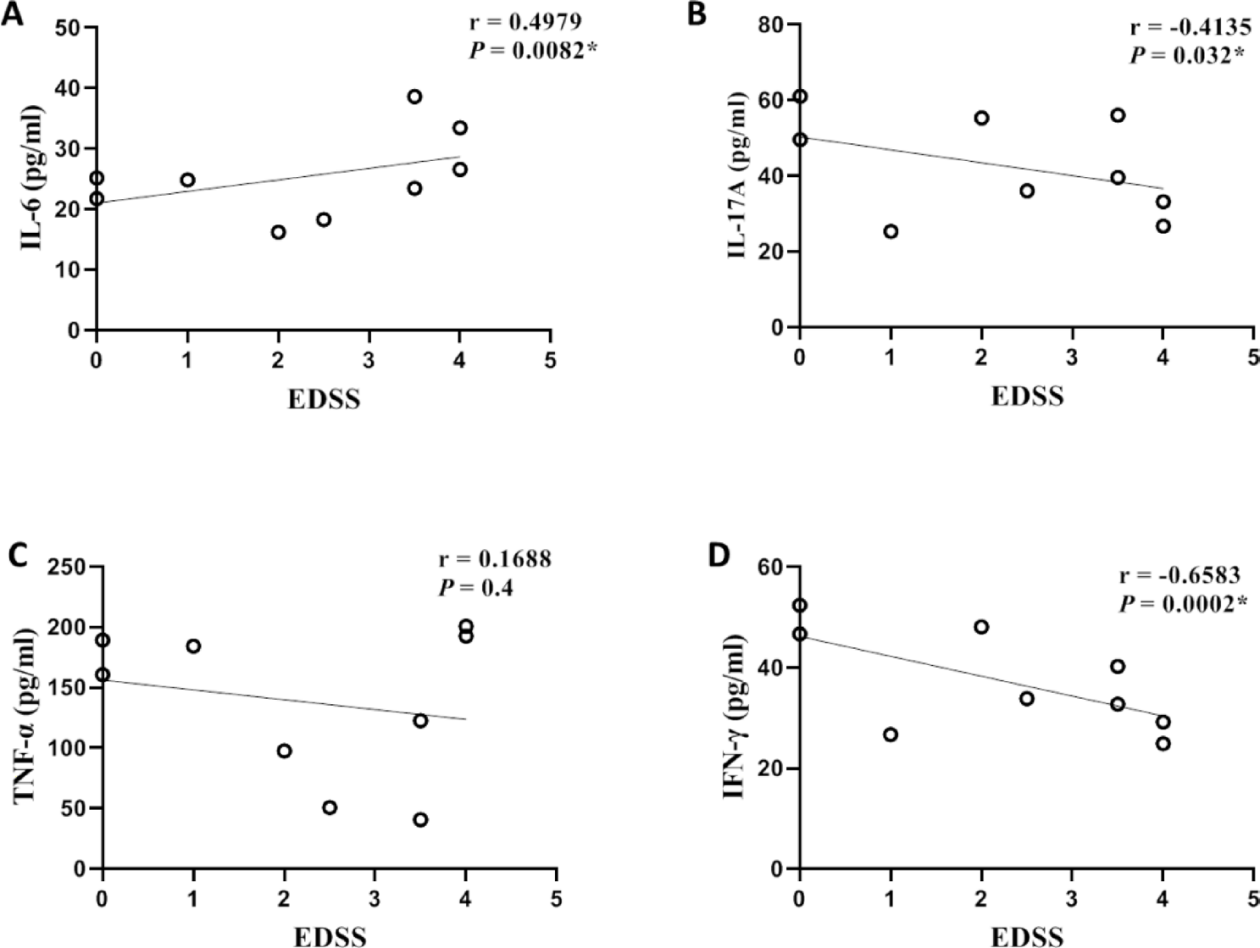
Linear correlation of serum cytokine levels and EDSS (n = 27). Scatter plots show the relationship between EDSS and (**A**) IL-6, (**B**) IL-17A, (**C**) TNF-α, and (**D**) IFN-γ. A significant positive correlation was observed between IL-6 and EDSS (r = 0.4979, *P* = 0.0082), while IL-17A and IFN-γ showed significant negative correlations with EDSS (r = –0.4135, *P* = 0.032; r = –0.6583, *P* = 0.0002, respectively). No significant association was detected between TNF-α and EDSS (r = 0.1688, *P* = 0.4). Correlations were assessed using Spearman’s correlation coefficient. **IL;** Interleukin**, TNF-α;** Tumor necrosis factor alpha**, IFN-γ;** Interferon gamma**, EDSS;** Expanded disability status scale**, r;** Spearman correlation coefficient, * indicates statistical significance (*P* < 0.05).

The correlation between cytokine levels and EDSS in treatment-naïve patients (n = 27) is shown in Fig. 2. IL-6 correlates directly (r = 0.4979, 95%CIs = 0.1338 to 0.7436, *P* = *0.0082*), while IL-17A and IFN-γ correlate inversely (r = -0.4135, − 0.6583, 95%CIs = − 0.6920 to − 0.02796, − 0.8342 to − 0.3609, *P* = *0.032, 0.0002*, respectively). Spearman rank correlations between serum cytokine pairs across treatment groups are provided in Table S5, while the correlation between cytokine levels and T2 lesion load is provided in Fig. S1.

#### Comparison of patient’s clinical status/parameters between MS patients with or without family history of autoimmunity

MS patients with a family history of autoimmunity [MS or other autoimmune disorders, mainly rheumatoid arthritis (RA) or systemic lupus erythematosus (SLE)] have a significantly earlier age at onset than patients without a family history (*P* < 0.0001) (Table 5). Additionally, MS patients with a family history of autoimmune disease than MS have a lower age at onset and a higher EDSS and IgG index than MS patients with a family history of MS (Table S7).

**Table 5:** Comparison of patient’s clinical status/parameters between MS patients with or without family history of autoimmunity.

Clinical status/parameters	Patients with a family history of autoimmunity (n = 18)	Patients without a family history of autoimmunity (n = 60)	*P* value
	Median (IQR)		
EDSS	1.50 (1.125–3.375)	2.00 (1.0–3.5)	0.3776
IgG index	0.7100 (0.33–1.27)	0.8950 (0.65–1.095)	0.2438
OCB	18.00 (17.0–19.0)	18.50 (8–22.0)	0.5896
Age at onset	17.00 (16.0–25.0)	29(23.0–32)	< 0.0001*

EDSS; Expanded disability status scale**, **OCB; Oligoclonal band**.**

* indicates statistical significance (*P* < 0.05) among groups.

[Mann–whitney test using exact *P* value]

**Fig. 3:**
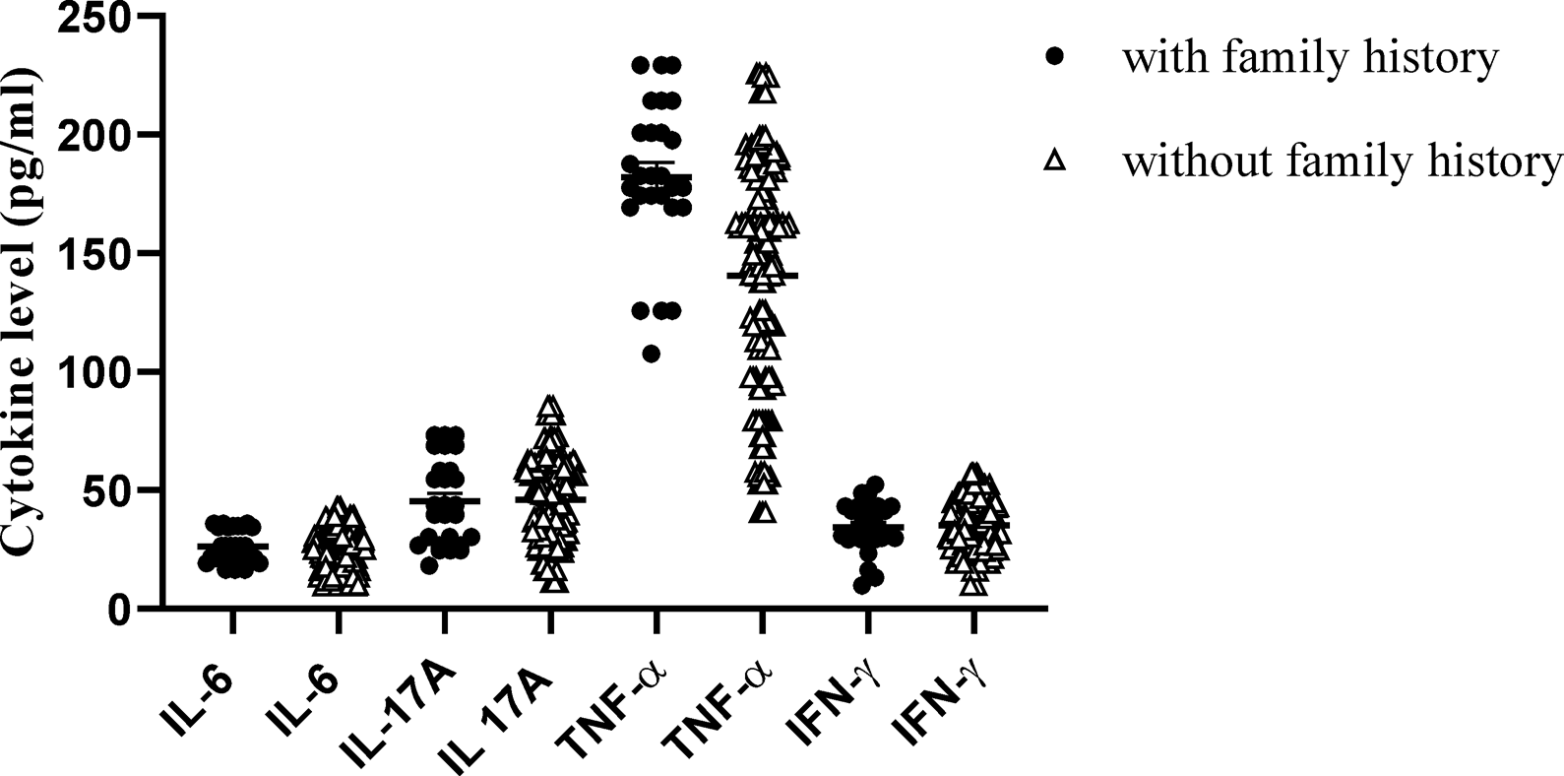
Comparison of serum cytokine levels between MS patients with or without family history of autoimmunity. IL; Interleukin, TNF-α; Tumor necrosis factor alpha, IFN-γ; Interferon gamma. [Mann–whitney test using adjusted *P* value].

Additionally, TNF-α is the only cytokine that showed a statistical difference in its level between MS patients with or without a family history of autoimmunity, with higher concentrations in patients with a family history (*P* = 0.0001; Fig. 3**)**.

## Discussion

In this study, we investigated the impact of different treatment durations with multiple DMTs on serum cytokine levels in MS patients and demonstrated a correlation between serum cytokine levels and patients’ clinical status/parameters, trying to shed light on the potential use of serum cytokine levels as a diagnostic or prognostic tool in MS.

The cytokines assessed in this study were strategically selected to represent pivotal mediators of the Th1 and Th17 immunological pathways, both of which are critically implicated in the pathogenesis and progression of MS ^33,34^. Their inclusion was further justified by the availability of highly sensitive and specific commercial assay platforms, facilitating standardized, rapid, and reproducible quantification. Additionally, the extensive evaluation of these cytokines in prior investigations provides a robust comparative framework, thereby enhancing the interpretability and external validity of our findings^10,35–37^. Using serum samples instead of CSF samples is more suitable for large-scale studies, as it is a less invasive and more cost-effective method^38^ and reflects the systemic inflammatory status of the disease^39^.

The patients were classified into untreated patients or treated patients, with the latter group further stratified by treatment duration (less than 12 months or more than 24 months). This classification was according to the guideline that stated that the full clinical effect of DMTs may take 6 months after drug initiation^40^. Also, the therapeutic response evaluation is held within the first 2 years after DMT initiation and recommends switching treatment in the presence of a suboptimal response^40^. Another guideline recommends switching DMT after 1 year of initiation^41^. Moreover, several studies investigated the effect of DMT on ameliorating the clinical features of disease, which takes over 12 months, and after 24 months^42,43^.

In this cohort, the level of all cytokines showed a trend toward increase in patients treated for less than 12 months compared to untreated patients, while, except for IL-17A, they decreased markedly in patients treated for more than 24 months. Supporting this observation, other studies have reported a non-significant change in IL-17A levels after 1–2 years of IFN-β treatment (various types/doses) relative to controls^44^, while IL-6 and TNF-α levels show a significant reduction after 24 months of therapy^45^. Another study demonstrated that long-term DMF treatment (> 18 months), but not short-term treatment (4–6 months), significantly reduced CXCR3 + CD4 + (Th1) and CCR6 + CD4 + (Th17) cells, aligning with reductions in IFN-γ and IL-17 producing T cells^46^. A similar reduction was also observed after 12 months of treatment^47^. Moreover, another study demonstrated a significant decrease in TNF-α level in patients treated with IFN-β after a period longer than 1 year^48^. Fingolimod treatment for 6 months resulted in an increase of IFN-γ and TNF-α producing CD4^+^ T cells, with a non-significant effect on IL-6, IL-17A, and TNF-α levels^49^.

Another study showed a non-significant change in serum IL-6, IL-17A, TNF-α, and IFN-γ levels in RRMS-treated patients for 2 months compared to untreated patients^36^. There was a non-significant change in cytokine mRNA-expressing PBMCs, including IFN-γ and TNF-α, through treatment with IFN-β for 2–9 months^50^. Additionally, a previous study showed that rituximab treatment for 4 months was associated with a non significant increase in Th1 cytokines production (TNF-α, IFN-γ) and a significant increase in Th2 and Th17 (IL-6, IL-17) cytokines at 16 weeks of treatment^34^. Another study showed a significant decrease in serum IL-6 and TNF-α levels by rituximab treatment at 5–12 weeks, and then their levels returned gradually to the baseline level at 36–48 weeks after treatment^51^. The early effect may be explained by the removal of cytokine-producing B-cells^52^, while increasing cytokine levels is attributed to the loss of the suppressive or regulatory effect of B cells through regulatory cytokine production on effector T cells and other innate cells^53,54^. On the other hand, there was a non-significant change of IL-6, TNF-α, and IFN-γ after 6 months of IFN-β^55^, treatment with INF-β decreases IL-17 significantly with a non-significant effect on INF-γ^56^. As shown, there are substantial discrepancies between studies that reflect the complexity of immune system pathways and responses against different DMTs. A further barrier to comprehending the possible mechanisms behind this investigation is the lack of studies tracking cytokine levels over extended time periods. Therefore, further investigations that track cytokine levels over periods exceeding 24 months could offer greater clarity regarding the biological mechanisms and the molecular processes behind therapy responsiveness. Additionally, careful observation of the clinical parameters and MRI disease activity along with cytokine levels will provide deeper insights into the potential of using cytokine profiling as a guide for personalized treatment decisions or to serve as a biomarker to monitor therapy effectiveness in MS patients.

In this cohort, only IL-6 was correlated directly with EDSS, while IL-17A and IFN-γ correlated inversely and TNF-α showed a non-significant correlation with EDSS. There is a significant negative correlation between IL-17 and IFN-γ with EDSS^36^ and a non-significant correlation between TNF-α and EDSS^57^. Numerous studies observed that the elevated IL-6 level is correlated with disability and deterioration in MS patients^35,58^. Also, a significant direct correlation of IL-6 level and EDSS was observed in patients with another autoimmune disorder that affects the spinal cord with the optic nerve called neuromyelitis optica spectrum disorder (NMOSD)^59–61^. In addition, IL-6 level is correlated with clinical parameters of inflammation in RA patients that are not achieved with TNF-α or IFN-γ^62^, while the non-significant correlation of IL-6 with EDSS in NMO patients is also observed^63^.

Our finding showed that MS patients with a family history of autoimmunity have a significantly lower age at onset than those MS patients without a family history. It was shown previously that there was a significantly lower age at onset in the familial MS versus the sporadic MS populations^64,65^. This observation suggests that there is a genetic burden that may contribute to a shorter preclinical MS period and a lower age at onset^64^. Some studies have reported an association between the human leukocyte antigen HLA-DRB1*1501 haplotype, a known familial risk factor, and a lower age at disease onset^66–68^. There were no significant differences in other clinical parameters between MS patients with a family history of autoimmunity and MS patients without a family history^64,69^. As shown in the current study, TNF-α was the only cytokine significantly elevated in patients with a family history of autoimmunity (MS, RA, SLE). This common link may be explained by shared HLA genetic variants or other major genetic risk factors at the HLA locus^70,71^. The TNF-α gene is encoded in the HLA region within the HLA III region in chromosome 6p21^72,73^, between HLA-B and HLA-DR^74^. This may explain the association of higher level of TNF-α in patients with a family history of MS and other autoimmune diseases. A study showed that the most common haplotype in familial MS is DRB1*1501, which is one of the HLA-DR2 haplotypes^68^. Another study observed increased production of TNF-α and lymphotoxin-α by CD4^+^ T-cell lines (TCLs) isolated from HLA-DR2^+^ carriers of MS patients without change in IFN-γ level^75^. Therefore, the HLA association of MS may be related to the production of TNF-α and lymphotoxin-α, an issue that needs additional work to confirm this relation.

To the best of our knowledge, few studies have comprehensively examined the impact of DMTs on cytokine profiles over extended periods. A major strength of this study lies in the inclusion of long-term treated patients (> 24 months), which provides valuable insights into the sustained immunological effects of these therapies. Unlike most prior investigations that focused on single time points or limited cytokine subsets, this research integrates a broader cytokine panel representing both Th1 and Th17 pathways and correlates these immune profiles with clinical parameters and patients’ history of autoimmunity.

The observation of altered cytokine dynamics in patients with a family history of autoimmunity may highlight a potential genetic or immunological predisposition, addressing a key gap in literature. Furthermore, the use of serum samples rather than CSF offers a less invasive, scalable, and clinically applicable approach, enhancing the potential for translation into routine disease monitoring.

Collectively, these strengths make this study a significant step toward developing accessible, blood-based biomarkers to guide therapeutic decisions and personalize treatment strategies in MS.

## Study limitations and future prospective 

The main limitations of the study include its cross-sectional design, the relatively small sample sizes within specific treatment subgroups, and the lack of quantitative MRI markers, such as CVS and PRLs, which were not part of the diagnostic protocol at the time of data collection. Although the absence of these imaging markers in our dataset does not compromise the validity of our results. Despite the inclusion of DMT class and sex in univariate analyses, with adjustment for age, disease duration, and clinical phenotype, the present study cannot fully disentangle the independent effect of treatment duration from the influence of the predominant DMT classes, particularly among individuals receiving long-term therapy. Consequently, associations attributed to prolonged treatment exposure should be interpreted with appropriate caution. In addition, circulating cytokine levels are subject to modulation by several factors that were not systematically captured in this study, including smoking status, body mass index, vitamin D supplementation, intercurrent infections, comorbid conditions, and concomitant symptomatic treatments, which may have contributed to residual confounding. Finally, the hypothesized relationship between HLA background and enhanced TNF-α production in MS patients with a family history of autoimmunity remains exploratory and requires validation in adequately powered genetic and functional studies.

Future investigations should aim to validate these findings in larger cohorts by classifying patients based on the type of DMTs used and correlating serum cytokine levels with treatment type and duration. The Inclusion of CVS and PRLs in correlations with cytokine levels and other clinical parameters, although not obligatory for MS diagnosis, may enhance the understanding of their combined contribution to disease activity and progression. Finally, examine a wider spectrum of pro-inflammatory and regulatory mediators, and explore gene polymorphisms in cytokine pathways to better elucidate mechanisms of treatment response and optimize individualized therapy.

## Conclusion

Various DMTs modulate cytokine levels over different treatment periods to exert their beneficial effect. In the present study, significant reductions in IL-6, TNF-α, and IFN-γ were evident only after prolonged treatment durations exceeding 12 months, whereas IL-17A levels did not demonstrate a statistically significant decline even with extended therapy. Furthermore, while cytokine levels could not serve as predictors of lesion load, IL-6 can be used as a prognostic factor of progression and disability status in MS. The elevated TNF-α observed in MS patients with a family history of MS may potentiate the role of HLA complex gene associations in MS pathogenesis.

## Supplementary Information

Below is the link to the electronic supplementary material.

Supplementary Material 1

## Data Availability

The datasets used and/or analyzed during the current study are available from the corresponding author on reasonable request.
