# Supplementary material for: Association of cytokine levels with treatment duration and patient family history in Egyptian multiple sclerosis patients
Source: Sci Rep. 2026 Mar 2;16:7951. doi: 10.1038/s41598-026-38500-z (PMC12957492; doi:10.1038/s41598-026-38500-z)
Supplement: Supplementary file 1 — Supplementary Material 1 [file 41598_2026_38500_MOESM1_ESM.docx]

**Table S1: Correlation of serum cytokines between treatment groups of RRMS patients.** In RRMS patients with different treatment durations, the level of IL-6 and IFN was significantly reduced in long treated than untreated or short treated (*P* =<0.0001, 0.0016, respectively), the TNF level was significantly increased in short treated than untreated patients (*P* = 0.0441), and significantly reduced in long treated than short treated (*P* =0.003). There is non-significant difference in IL-17A level. (**Table S1**)

| **Serum cytokines** | **RRMS** | | | |
| --- | --- | --- | --- | --- |
|  | **non-treated (Treatment gps 1, 3)** | **short treated (Treatment gp 2)** | **long treated (Treatment gp 4)** | ***P* value** |
|  | Median  (IQR) | | |  |
| **IL-6** | 26.59  (21.76-33.14) | 25.21  (21.76-34.52) | ******#16.59  (11.76-22.10) | <0.0001* |
| **IL-17A** | 43.93  (26.79-58.93) | 54.64  (31.79-58.93) | 48.21  (36.07-61.79) | 0.1887 |
| **TNF-α** | 160.8  (119.2-180.8) | **^@^**175.8  (125.8-199.2) | ******140.8  (57.50-175.8) | 0.003* |
| **IFN-γ** | 33.86  (29.57-46.71) | 33.86  (29.93-48.86) | ******#25.29  (19.21-42.79) | 0.0016* |
| **^@^** indicates significance between Treatment gp 1,3 Vs. Treatment gp 2 **(Dunn’s test).**  # indicates significance between Treatment gp 1,3 Vs. Treatment gp 4 **(Dunn’s test).**  ****** indicate significance between Treatment gp 2 Vs. Treatment gp 4 **(Dunn’s test).**  * **i**ndicates statistical significance (*P* < 0.05).  **IL; Interleukin, TNF- α; Tumor necrosis factor alpha, IFN-γ; Interferon gamma, IQR;** Interquartile range (Q1-Q3)**.**  [kruskal-wallis test with approximate P value/Dunn's multiple comparisons test using adjusted *P* value]  ***P* values represent overall differences among the three groups assessed by the Kruskal–Wallis test; post-hoc pairwise comparisons (Dunn’s test) are not shown.** | | | | |

**Table S2: Correlation of serum cytokines between treatment groups of SPMS patients**. In SPMS patients with different treatment durations, the level of IL-6 was significantly reduced in long treated than non-treated (*P* =<0.0426), the TNF level was significantly reduced in long treated than non-treated or short treated (*P* =0.0017). There is non-significant difference in IL-17A or IFN- levels. (**Table S2**)

| **Serum cytokines** | **SPMS** | | | |
| --- | --- | --- | --- | --- |
|  | **non-treated (Treatment gps 1, 3)** | **short treated (Treatment gp 2)** | **long treated (Treatment gp 4)** | ***P* value** |
|  | Median  (IQR) | | |  |
| **IL-6** | 25.21  (21.41-30.72) | 26.59  (23.94-30.22) | #16.24  (13.48-19.69) | 0.0314* |
| **IL-17A** | 42.50  (16.07-51.79) | 71.79  (61.14-78.39) | 35.71  (28.21-69.11) | 0.0897 |
| **TNF-α** | 160.8  (149.2-175.8) | 185.8  (152.9-193.2) | ******#92.50  (79.17-120.8) | 0.0017* |
| **IFN-γ** | 32.79  (30.29-46.36) | 43.14  (36.57-46.57) | 38.14  (28.50-38.86) | 0.1347 |
| #**;** indicates significance between Treatment gp 1,3 Vs. Treatment gp 4 **(Dunn’s test).**  ****;** indicate significance between Treatment gp 2 Vs. Treatment gp 4 **(Dunn’s test).**  *; **i**ndicates statistical significance (*P* < 0.05).  **IL;** Interleukin, **TNF- α;** Tumor necrosis factor alpha**, IFN-γ;** Interferon gamma**, IQR;** Interquartile range (Q1-Q3)**.**  [kruskal-wallis test with approximate *P* value/Dunn's multiple comparisons test using adjusted *P* value]  ***P* values represent overall differences among the three groups assessed by the Kruskal–Wallis test; post-hoc pairwise comparisons (Dunn’s test) are not shown.** | | | | |

**Table S3: Serum cytokine levels of the treatment groups.** Median concentrations (pg/mL) and interquartile ranges (IQR) of IL-6, IL-17, TNF-α, and IFN-γ measured in serum samples collected from Treatment Groups 1–4. Values reflect the systemic inflammatory response associated with each treatment condition.

|  | **Treatment gp 1** | **Treatment gp 2** | **Treatment gp 3** | **Treatment gp 4** |
| --- | --- | --- | --- | --- |
|  | Median (pg/ml)  (IQR) | | | |
| **IL-6** | 24.86  (21.5-30.55) | 25.90  (21.84-33.48) | 28.14  (23.57-34.69) | 16.41  (13.22-21.5) |
| **Il-17** | 38.93  (28.21-55.89) | 56.07  (33.75-66.43) | 43.93  (20.18-61.07) | 47.86  (35.0-61.61) |
| **TNF-α** | 152.5  (102.9-190.4) | 176.7  (128.7-198.8) | 161.7  (146.7-169.2) | 106.7  (68.33-172.1) |
| **IFN-γ** | 33.14  (29.30-47.79) | 34.75  (30.20-47.43) | 38.68  (30.55-46.36) | 29.04  (21.27-42.43) |
| **IL;** Interleukin, **TNF- α;** Tumor necrosis factor alpha**, IFN-γ;** Interferon gamma**, IQR;** Interquartile range (Q1-Q3). | | | | |

**Table S4: correlation of serum cytokine levels between treatment groups.** Differences in cytokine concentrations (IL-6, IL-17, TNF-α, and IFN-γ) were analyzed between all treatment group pairs (Gp1–Gp4). Reported values include the mean rank differences and their corresponding *p*-values. Significant differences are indicated by an asterisk (*). Analyses were performed using the Kruskal–Wallis test with approximate *p*-values, followed by Dunn’s multiple comparisons test with adjusted *p*-values.

|  | **diff.**  **(Gp1 vs.Gp2)**  **p value** | **diff.**  **(Gp1 vs. Gp3)**  **p value** | **diff.**  **(Gp1 vs. Gp4)**  **p value** | **diff.**  **(Gp2 vs.Gp3)**  **p value** | **diff.**  **(Gp2 vs. Gp4)**  **p value** | **diff.**  **(Gp3 vs.Gp4)**  **p value** |
| --- | --- | --- | --- | --- | --- | --- |
| **Il-6** | **0.2249** | **0.0519** | **<0.0001*** | **0.2656** | **<0.0001*** | **<0.0001*** |
| **Il-17** | **0.0247*** | **0.6030** | **0.3429** | **0.0247*** | **0.1895** | **0.2011** |
| **TNF-α** | **0.0320*** | **0.6974** | **0.0444*** | **0.0207*** | **<0.0001*** | **0.0014*** |
| **IFN-γ** | **0.6259** | **>0.9999** | **0.0053*** | **0.9782** | **0.0031*** | **0.0043*** |
| **IL;** Interleukin**, TNF- α;** Tumor necrosis factor alpha**, IFN-γ;** Interferon gamma**.**  * **i**ndicates statistical significance (*P* < 0.05).  [Mann-whitney test with Exact *P* value] | | | | | | |

**Table S5: Spearman rank correlations between serum cytokine pairs across treatment groups.** Spearman’s rank correlation coefficients (r) and corresponding *p*-values are presented for the relationships between IL-6, IL-17A, TNF-α, and IFN-γ within each treatment group (Gp1–Gp4). Only statistically significant correlations are shown. Positive and negative *r* values indicate direct and inverse associations, respectively, reflecting how cytokine responses covary under different treatment conditions

| **Serum cytokines** | **Treatment Group** | **r** | ***P* value** |
| --- | --- | --- | --- |
| **IL-6/IL-17A** | **Treatment gp 1** | **0.357** | **0.013** |
| **IL-6/TNF-α** | **Treatment gp 3** | **-0.424** | **0.003** |
|  | **Treatment gp 4** | **0.555** | **0.0001** |
| **IL-6/IFN-γ** | **Treatment gp 4** | **0.314** | **0.03** |
| **IL-17A/TNF-α** | **Treatment gp 1** | **-0.302** | **0.037** |
|  | **Treatment gp 2** | **0.705** | **0.0001** |
| **IL-17A/IFN-γ** | **Treatment gp 1** | **0.893** | **0.0001** |
|  | **Treatment gp 2** | **0.839** | **0.0001** |
|  | **Treatment gp 3** | **0.622** | **0.0001** |
| **TNF-α/IFN-γ** | **Treatment gp 1** | **-0.328** | **0.023** |
|  | **Treatment gp 2** | **0.577** | **0.0001** |
|  | **Treatment gp 4** | **0.533** | **0.0001** |
| **IL;** Interleukin, **TNF- α;** Tumor necrosis factor alpha**, IFN-γ;** Interferon gamma**.**  **r;** spearman rank coefficient. | | | |

**Table S6. Comparison of clinical parameters between patients with relapsing–remitting multiple sclerosis (RRMS) and secondary progressive multiple sclerosis (SPMS).** Median values and interquartile ranges (IQR) are presented for EDSS at onset, EDSS at the time of sampling, number of relapses, age at onset, and IgG index in RRMS and SPMS patients. *P*-values indicate the significance of differences between groups, analyzed using the Mann–Whitney test with exact *p*-values.

| **Clinical Parameter** | **RRMS** | **SPMS** | ***P* value** |
| --- | --- | --- | --- |
|  | Median (IQR) | | |
| **EDSS at onset** | 2 (1.5-3.5) | 5.5 (4-6.5) | <0.0001* |
| **EDSS at sampling** | 3 (1-4) | 5.25 (4-6) | <0.0001* |
| **No. of relapses** | 1 (1-2) | 3.5 (2.25-7.25) | <0.0001* |
| **Age at onset** | 24 (19-31) | 30 (21.25-32.5) | 0.0221* |
| **IgG Index** | 0.8950  (0.68-.226) | 0.7200  (0.48-1.22) | 0.2989 |
| **RRMS;** Relapsing remitting multiple sclerosis, **SPMS;** Secondary progressive multiple sclerosis, **EDSS;** Expanded disability status scale, **IQR;** Interquartile range (Q1-Q3).  *; **i**ndicates statistical significance (*P* < 0.05).  [Mann-whitney test with Exact *P* value] | | | |

**Table S7.** **Comparison of clinical characteristics between MS patients with a family history of multiple sclerosis and those with a family history of other autoimmune diseases.**Median values and interquartile ranges (IQR) are presented for age at onset, EDSS score, IgG index, and CSF oligoclonal bands (OCBs). *P*-values indicate the significance of differences between groups, reflecting the potential influence of familial MS versus broader autoimmune predisposition on clinical presentation

| **Clinical parameters** | **Patients with family history of MS** | **Patients with family history of autoimmunity** | **P value** |
| --- | --- | --- | --- |
|  | Median (IQR) | |  |
| **Age at onset** | 25 (21-29) | 17 (16-17) | 0.0317* |
| **EDSS** | 1.25 (1-1.5) | 2 (1.5-4) | 0.0078* |
| **IgG index** | 0.33 (0.3325-0.3375) | 0.99 (0.71-1.27) | 0.0048* |
| **CSF-OCBs** | 19 (18.25-19.75) | 17 (16.25- 17.75) | 0.0571 |
| **EDSS;** Expanded disability status scale, **OCBs**; oligoclonal bands, **IQR;** Interquartile range (Q1-Q3).  *; **i**ndicates statistical significance (*P* < 0.05). [Mann-whitney test with Exact *P* value] | | | |

**Table S8. Immune ratio profiles (IL-6/TNF-α and IL-17A/IFN-γ) across the study treatment groups.** Median values and interquartile ranges (IQR) of the cytokine immune ratios IL-6/TNF-α and IL-17A/IFN-γ are presented for Treatment Groups 1–4. These ratios reflect the balance between pro-inflammatory cytokines within each treatment condition and provide insight into the overall immune response profile associated with each group**.**

| **Treatment Group** | **IL-6/TNF-α** | **IL17A/IFN-γ** | |
| --- | --- | --- | --- |
|  | Median (IQR) | | |
| **Treatment gp1** | 0.1727 (0.1337-0.3057) | | 1.151 (0.9776-1.377) |
| **Treatment gp2** | 0.1499 (0.121-0.2406) | | 1.424 (1.093-1.659) |
| **Treatment gp3** | 0.1740 (0.1301-0.2146) | | 1.258 (0.6825-1.368) |
| **Treatment gp4** | 0.1719 (0.105-0.2458) | | 1.483 (1.17-1.926) |
| **IL;** Interleukin, **TNF- α;** Tumor necrosis factor alpha**, IFN-γ;** Interferon gamma**, IQR;** Interquartile range (Q1-Q3). | | | |

Concerning the correlation of cytokines level and the T2 lesions load in MRI of MS patients, there wasn’t any significance association of any tested cytokine level with the T2 lesions load (*P* >0.05) (**Fig S1**).

**Figure S1. Correlation between serum cytokines level and T2 lesions load.**

Bar graphs illustrate the concentrations of IL-6, IL-17A, TNF-α, IFN-γ (pg/ml) stratified by T2 lesions load (1-3, 4-9, ≥10) lesions), with non-significant difference of any cytokine level between T2 lesions load. **IL**; Interleukin, **TNF- α**; Tumor necrosis factor alpha**, IFN-γ**; Interferon gamma.
